# Decentralization of viral load testing to improve HIV care and treatment cascade in rural Tanzania: observational study from the Kilombero and Ulanga Antiretroviral Cohort

**DOI:** 10.1186/s12879-023-08155-6

**Published:** 2023-04-07

**Authors:** Dorcas Mnzava, James Okuma, Robert Ndege, Namvua Kimera, Alex Ntamatungiro, Amina Nyuri, Theonestina Byakuzana, Faraji Abilahi, Paul Mayeka, Emmy Temba, Teddy Fanuel, Tracy Renée Glass, Thomas Klimkait, Fiona Vanobberghen, Maja Weisser, on behalf of the KIULARCO Study Group, Aschola Asantiel, Farida Bani, Manuel Battegay, Theonestina Byakuzana, Adolphina Chale, Anna Eichenberger, Gideon Francis, Hansjakob Furrer, Tracy Renée Glass, Speciosa Hwaya, Aneth V. Kalinjuma, Bryson Kasuga, Andrew Katende, Namvua Kimera, Yassin Kisunga, Olivia Kitau, Thomas Klimkait, Ezekiel Luoga, Herry Mapesi, Mengi Mkulila, Margareth Mkusa, Slyakus Mlembe, Dorcas Mnzava, Gertrud J. Mollel, Lilian Moshi, Germana Mossad, Dolores Mpundunga, Athumani Mtandanguo, Selerine Myeya, Sanula Nahota, Regina Ndaki, Robert Ndege, Agatha Ngulukila, Alex Ntamatungiro, Amina Nyuri, James Okuma, Daniel H. Paris, Leila Samson, Elizabeth Senkoro, Jenifa Tarimo, Yvan Temba, Juerg Utzinger, Fiona Vanobberghen, Maja Weisser, John Wigayi, Herieth Wilson, Bernard Kivuma, George Sigalla, Ivana Di Salvo, Michael Kasmiri, Suzan Ngahyoma, Victor Urio, Aloyce Sambuta, Francisca Chuwa, Swalehe Masoud, Yvonne R. Haridas, Jacqueline Nkouabi

**Affiliations:** 1 Ifakara Health Institute, Ifakara Branch, Ifakara, United Republic of Tanzania; 2 Swiss Tropical and Public Health Institute, Basel, Switzerland; 3 University of Basel, Basel, Switzerland; 4 St. Francis Referral Hospital, Ifakara, United Republic of Tanzania; 5 USAID Boresha Afya, Morogoro, United Republic of Tanzania; 6 Department of Biomedicine, Molecular Virology, University of Basel, Basel, Switzerland; 7 Division of Infectious Diseases and Hospital Epidemiology, University Hospital Basel, CH, Basel, Switzerland

**Keywords:** HIV cascade, Viral load testing, Viral suppression, Failure, Low level viremia

## Abstract

**Introduction:**

Monitoring HIV viral load (HVL) in people living with HIV (PLHIV) on antiretroviral therapy (ART) is recommended by the World Health Organization. Implementation of HVL testing programs have been affected by logistic and organizational challenges. Here we describe the HVL monitoring cascade in a rural setting in Tanzania and compare turnaround times (TAT) between an on-site and a referral laboratory.

**Methods:**

In a nested study of the prospective Kilombero and Ulanga Antiretroviral Cohort (KIULARCO) we included PLHIV aged ≥ 15 years, on ART for ≥ 6 months after implementation of routine HVL monitoring in 2017. We assessed proportions of PLHIV with a blood sample taken for HVL, whose results came back, and who were virally suppressed (HVL < 1000 copies/mL) or unsuppressed (HVL ≥ 1000 copies/mL). We described the proportion of PLHIV with unsuppressed HVL and adequate measures taken as per national guidelines and outcomes among those with low-level viremia (LLV; 100–999 copies/mL). We compare TAT between on-site and referral laboratories by Wilcoxon rank sum tests.

**Results:**

From 2017 to 2020, among 4,454 PLHIV, 4,238 (95%) had a blood sample taken and 4,177 (99%) of those had a result. Of those, 3,683 (88%) were virally suppressed. In the 494 (12%) unsuppressed PLHIV, 425 (86%) had a follow-up HVL (102 (24%) within 4 months and 158 (37%) had virologic failure. Of these, 103 (65%) were already on second-line ART and 32/55 (58%) switched from first- to second-line ART after a median of 7.7 months (IQR 4.7–12.7). In the 371 (9%) PLHIV with LLV, 327 (88%) had a follow-up HVL. Of these, 267 (82%) resuppressed to < 100 copies/ml, 41 (13%) had persistent LLV and 19 (6%) had unsuppressed HVL. The median TAT for return of HVL results was 21 days (IQR 13–39) at the on-site versus 59 days (IQR 27–99) at the referral laboratory (*p* < 0.001) with PLHIV receiving the HVL results after a median of 91 days (IQR 36–94; similar for both laboratories).

**Conclusion:**

Robust HVL monitoring is achievable in remote resource-limited settings. More focus is needed on care models for PLHIV with high viral loads to timely address results from routine HVL monitoring.

## Introduction

The number of people living with HIV (PLHIV) on antiretroviral therapy (ART) globally and in Tanzania has been increasing over time with 82% accessing ART in 2020 [1]. HIV viral load (HVL) testing in PLHIV – the preferred monitoring approach by the World Health Organization (WHO) [2] – allows early detection of treatment failure and guides treatment decisions, for example adherence counselling or regimen change. In sub–Saharan Africa, HVL monitoring has been scaled up in recent years with investments strengthening HVL processing hubs and sample transportation systems, to overcome challenges of transport and result transmission especially in remote settings [3]. In Tanzania, the government scaled up routine HVL testing in 2017 through establishment of HVL processing centres (hubs) and policies enabling all PLHIV to receive HVL testing [4, 5]. For remote health facilities, the long distances to referral HVL processing hubs, poor road infrastructures (especially during the rainy season), unreliable transport systems, and the danger of blood sample degradation due to failure of cold chains remain a challenge [6, 7].

For successful HVL monitoring, a reliable testing process with a rapid turnaround time (TAT) is key and affects all stages of the testing cascade including blood withdrawal, sample transport and processing, generation of results, feedback to clinicians and PLHIV, and timely subsequent action. Samples for HVL testing are recommended to be processed and results made available to the clinicians within 14 days for management of treatment [8]. According to the Tanzania National AIDS Control Program guidelines, the first HVL test is done at 6 months after initiation of ART and a second at 12 months. If both viral load results are < 1000 copies/mL, HVL is then monitored yearly. PLHIV with unsuppressed HVL receive enhanced adherence counselling for three months followed by a follow-up HVL test in the fourth month—if reported adherence is > 95%. If the follow-up HVL result is below 1000copies/ml, the patient will continue with the same drug regimen, otherwise the recommendation is to switch to a second-line ART regimen [8].

Gaps in the HVL cascade, such as long TAT, delay in timely initiation of adherence counselling and/or switch to second-line ART in PLHIV with virologic failure (VF) lead to an increased risk of accumulation of drug resistance mutations, development of clinical failure and mortality as well as onwards HIV transmission [9–11].

We hypothesised that decentralization of HVL testing from a referral laboratory to an on-site molecular laboratory capable of conducting HVL testing, hence avoiding sample transport to a central referral laboratory, will reduce TAT of HVL results. Therefore, we aimed at analysing the HVL monitoring cascade, comparing turnaround times of viral load results when viral load testing was done at an on-site laboratory versus a referral laboratory. We furthermore assessed management and outcome among those with high- and low-level viremia following the national rollout of routine HVL monitoring in a cohort of PLHIV in rural Tanzania.

## Methods

### Study setting

This is an observational study in the prospective Kilombero and Ulanga Antiretroviral Cohort (KIULARCO). Kilombero and Ulanga are rural districts in the Morogoro region in South-western Tanzania, with St. Francis Referral Hospital (SFRH) being the largest health facility providing HIV care and treatment in the region. Morogoro Regional Referral Hospital (MRRH) – used for referral of laboratory services – is located 229 kms (km) away from SFRH. KIULARCO has existed since 2005 as collaboration between the SFRH, the Ifakara Health Institute (IHI), the Swiss Tropical and Public Health Institute, and the University Hospital of Basel, Switzerland. PLHIV attending the Chronic Diseases Clinic of Ifakara (CDCI) of the SFRH are invited to participate in KIULARCO. Almost 11,000 PLHIV have been enrolled in KIULARCO as of January 2021, with information collected prospectively in an electronic patient records database (openMRS), including demographics, clinical and laboratory data, medication history, drug toxicities, diagnoses and outcomes. Details of the cohort have been described elsewhere [12, 13].

Routine HVL testing at SFRH was implemented in August 2017. Plasma samples from PLHIV were transported to the referral laboratory at MRRH for analysis. Additionally, a point of care system (GeneXpert ®) for HVL testing was operating at the on-site laboratory with a 2-h TAT for urgent cases. With the aim to decentralize HVL testing, from August 2018 onwards, HVL testing was started at the on-site laboratory at IHI in close collaboration with the National AIDS Control Program and the implementing partner USAID Boresha Afya. On-site laboratory at IHI offered HVL testing for SFRH patients and was referral laboratory for HVL testing for other peripheral health facilities. Referral of samples to MRRH was then only done in case of stock outs of reagents or machine breakdown.

### Study population

For this study, we included all PLHIV with an informed consent enrolled in KIULARCO, who were at least 15 years old and on ART for at least 6 months with a clinical visit from August 2017 to July 2020, with follow-up through January 2021.

### HVL testing and reporting procedures

During routine visits, venous blood samples were collected in BD vacutainer EDTA tubes® by the phlebotomist at the clinics and brought to the on-site laboratory the same day. In the first year of roll out of HVL testing (August 2017 to July 2018), blood samples were immediately centrifuged at the on-site laboratory and plasma was stored at -80 °C freezer until transport to MRRH for HVL testing. Transportation of the frozen plasma happened mostly twice a week. Upon arrival of the samples at MRRH, data clerks registered the patient-related information from the HVL request forms and, depending on the work load, the samples were processed immediately or stored in -20 °C freezers until they could be processed. HVL testing was done using COBAS® AmpliPrep/COBAS® TaqMan® system, according to manufacturers’ instructions with lowest detection limit of 20 copies/ml. Paper forms with the HVL results were brought from the MRRH to the SFRH by staff who carried the samples from SFRH to the MRRH or by local arrangements with office cars whenever possible. Data clerks entered the results in the openMRS database and filed paper copies in the patient files.

From August 2018 to July 2020, HVL testing happened at IHI, at the on-site laboratory on an Abbott m2000 System as per manufacturer’s instructions with lowest detection limit of 40copies/ml or 150copies/ml depending on the manufacturer’s protocol used. Depending on the work load, samples were processed immediately or stored in -80 °C freezers until processed. HVL results were then entered in the openMRS database. PLHIV were scheduled for routine HVL testing or follow-up of unsuppressed HVL following national guidelines [8]. In addition to HVL control according guidelines, we controlled HVL in PLHIV with LLV at their next scheduled visit.

### Definitions

We defined baseline as the first clinical visit of eligible patients. The HVL measurement at this time is referred to as the first HVL. We considered a HVL ≥ 1000copies/mL as ‘unsuppressed’ and a HVL < 1000copies/mL as ‘suppressed’. Virological failure (VF) was defined as two consecutives unsuppressed HVL results despite adherence counselling. Due to having different HVL measuring platforms with different lowest detection limits, we considered a HVL of 100 copies/ml as the lower limit for HVL detection. Hence LLV was defined as HVL 100–999 copies/mL, and persistent LLV as two consecutive LLV.

**Fig. 1:**
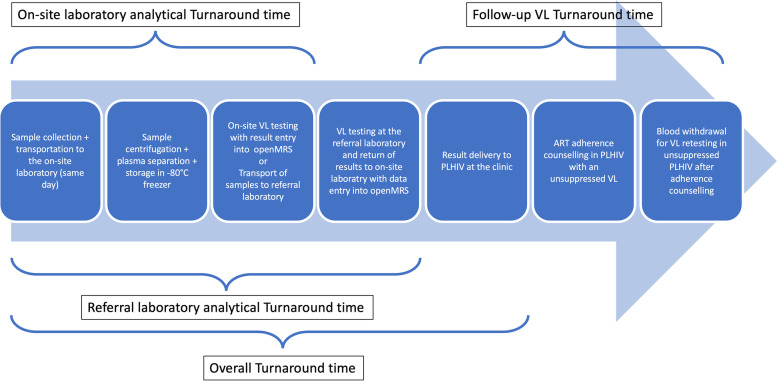
Schematic diagram for analytical, overall and follow-up TAT

The analytical TAT was defined as the number of days elapsed between the date a sample was collected until the date the HVL result was entered in the OpenMRS database and thus available to the clinician. Between these time points is the transport to the laboratory, the processing of the sample, result generation, result return to the on-site laboratory (if sent to the referral laboratory) and entry into the openMRS database. The overall TAT was defined as the number of days elapsed between the date a sample was collected until the date the patient received the results (among those who returned) and therapeutic measures could be taken (first clinical visit after the result was entered into OpenMRS). Additionally, for PLHIV with unsuppressed HVL and a follow-up HVL, the follow-up TAT was defined as the number of days elapsed between the date of unsuppressed HVL result entry in the openMRS database to the date follow up HVL result was entered in openMRS, Fig. 1.

Analytical TAT defined as the number of days elapsed between date of a sample was collected to date of HVL result entry into OpenMRS with immediate availability to the clinician. Overall, TAT defined as the number of days elapsed between date a sample was collected to date the patient received results (corresponding to the next clinical visit among those who returned) and therapeutical consequences could take place. For patients with unsuppressed HVL and a follow-up HVL, follow up TAT defined as the number of days elapsed between date of unsuppressed HVL result entry into openMRS database and date follow up HVL is entered in openMRS.

### Covariates

Independent baseline covariates considered were age, gender, marital status, highest education level, occupation, distance in km of residence from the clinic, daily alcohol consumption, body mass index (BMI), HIV WHO stage, CD4 cell count, ART regimen line, years since ART initiation, and calendar year. BMI, CD4 cell count and HIV WHO stage were those measurements closest to baseline, provided within 18 months before and up to 3 months after.

### Statistical methods

Medians, interquartile (IQ) ranges, frequencies and proportions were used to describe PLHIV’ baseline characteristics, the HVL testing cascade and TAT. We describe the proportion of PLHIV with sample taken for HVL testing among all participants, and of these, the proportion with reported HVL results. Following the first HVL measurement, we describe the cascade i) proportions of those virally suppressed ii) unsuppressed HVL and iii) LLV. In each group we report on the TAT of results and compared TAT results between the on-site and referral laboratories using Wilcoxon rank sum tests of medians for continuous variables. We used two-sided p-values for all tests. Analyses were performed using Stata version 15 [14].

## Results

### Baseline characteristics

From August 2017 to July 2020, 5,561 PLHIV visited the clinic. Of these, 1,107 were excluded from the analysis due to age < 15 years (*N* = 425), not being on ART (*N* = 89) or on ART for less than 6 months (*N* = 593 Among the 4,454 PLHIV included, 3,082 (69%) were female and median age was 42 years (IQR 35–51; Table 1). More than half of PLHIV (*N* = 2,528; 57%) were married, 3,709 (84%) had primary school education, 3,666 (83%) were farmers and 2,074 (48%) lived within a distance of less than 1 km from the clinic. The majority of PLHIV had a normal body mass index of 18.5- 25 kg/m^2^ (2,582; 60%). A WHO stage III/IV was diagnosed in 1,998 (45%) and the CD4 cell count was < 350 cells/ml in 1,205 (27%). The majority of PLHIV (2,798 (63%)) had been on ART for more than 2 years.

**Table 1:** PLHIV’ characteristics at baseline^a^

**Characteristics**	**All PLHIV** ***n* = 4454**
**Socio-demographics**	
Age categories (years), n (%)	
15 – 24	281 (7%)
25 – 34	769 (17%)
35—44	1546 (35%)
≥ 45	1858 (42%)
Gender, n (%)	
Male	1372 (31%)
Female	3082 (69%)
Married Status, n (%)	
Married/Cohabiting (%)	2528 (57%)
Never married	474 (11%)
Separated/divorced/widowed	1451 (33%)
Missing	1 (< 0.1%)
Education, n (%)	
None	363 (8%)
Primary	3709 (84%)
Secondary and above/other	371 (8%)
Missing	11 (0.3%)
Occupation, n (%)	
Farmer	3666 (83%)
Non farmer	777 (18%)
Missing	11 (0.3%)
Distance of residence from clinic, n (%)	
≤ 1 km	2074 (48%)
2 – < 50 km	1451 (33%)
≥ 50 km	835 (19%)
Missing	94 (2%)
Daily alcohol consumption, n (%)	
No	4403 (98%)
Yes	101 (2%)
**Clinical**	
Body Mass Index, Kg/m^2^, n (%)^b^	
Underweight, < 18.5	365 (8%)
Normal, 18.5—< 25	2582 (60%)
Overweight, 25—< 30	922 (21%)
Obese, ≥ 30	464 (11%)
Missing	121 (3%)
WHO Stage, n (%)^b^	
I	1463 (33%)
II	986 (22%)
III	1378 (31%)
IV	620 (14%)
Missing	7 (0.2%)
CD4 cell count (cells/µl)^b^, n (%)	
< 100	157 (4%)
100—349	1048 (24%)
≥ 350	3192 (73%)
Missing	57 (1%)
On Second line ART, n (%)	
No	3976 (89%)
Yes	478 (11%)
Baseline^a^ calendar year, n (%)	
2017	3045 (68%)
2018	813 (18%)
2019	465 (11%)
2020	131 (3%)
Years since ART initiation, n (%)	
< 2 years	1656 (37%)
2 – < 5 years	1100 (25%)
≥ 5 years	1698 (38%)

The characteristics analyzed are in number and percent of those with non-missing data, missing data rows are in number and column %

^a^At the first clinical visit aged ≥ 15 years old with at least 6 months on ART between August 2017 to July 2020

^b^Nearest recorded measurement of BMI, CD4 cell count, WHO stage 18 months before to 3 months after baseline

### Viral load testing cascade

**Fig. 2:**
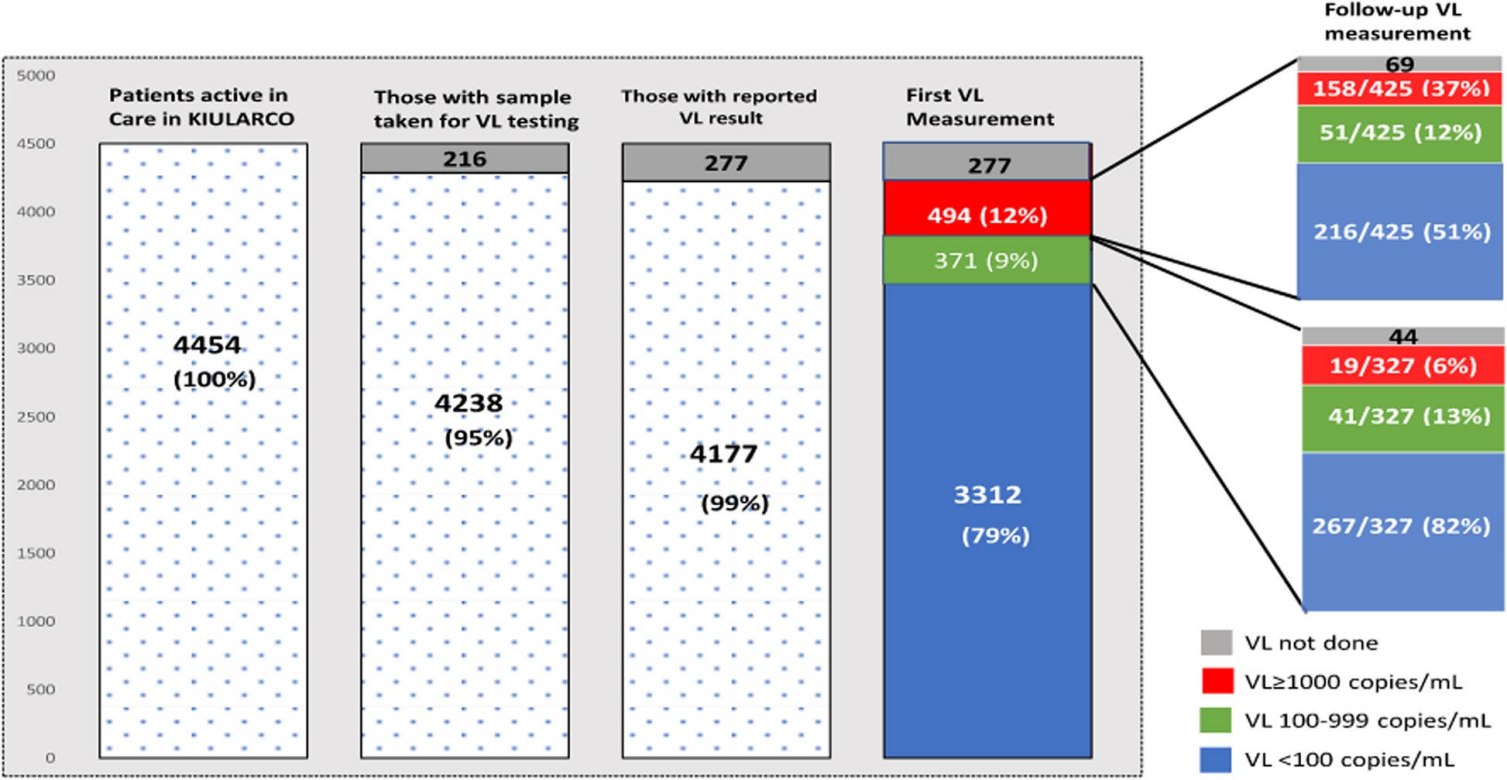
HIV viral load cascade. HIV viral load monitoring cascade of KIULARCO PLHIV (percentages are of the numbers in the preceding bar). Bars show number of patients at different stages of the HVL monitoring cascade

Among the 4,454 PLHIV, 4,238 (95%) had a blood sample taken for HVL measurement. Of those, 4,177 (99%) had a HVL result reported in the openMRS database (Fig. 2 and Table 2). Viral suppression was documented in 3,683/4,177 (88%) PLHIV, whereby 3,312 (79%) had a HVL < 100 copies/ml, 371 (9%) had LLV and 494 (12%) were unsuppressed.

**Table 2:** HIV Viral load monitoring cascade and outcomes

**Description**	**N (%) or Median (IQR)**
1. Cascade of viral load testing	
a. Number of PLHIV	4,454
b. PLHIV with blood sample taken for HVL testing	4,238/4,454 (95%)
c. PLHIV with a HVL result reported in the medical records	4,177/4,238 (99%)
i. Overall number of HVL test results	12,512
ii. HVL tests per person, median (IQR)	3 (2–4)
2. Results of first HVL measurement	
a. HVL < 1000 copies/mL (suppressed HVL)	3,683/4,177 (88%)
i. HVL 100–999 copies/mL (Low-level viremia (LLV))	371/4,177 (9%)
ii. HVL < 100 copies/mL	3,312/4,177 (79%)
b. HVL ≥ 1000 copies/mL (unsuppressed HVL)	494/4,177 (12%)
3. Cascade of care for PLHIV with HVL < 100 copies/mL, *n* = 3,312	
a. Number with follow-up HVL	2,876/3,312 (87%)
b. Median time to follow-up HVL, *n* = 2,876	12.0 months (11.8–13.2)
c. HVL < 100 copies/mL	2,653/2,876 (92%)
d. LLV	132/2,876 (5%)
e. Unsuppressed HVL	91/2,876 (3%)
4. Cascade of care for PLHIV with LLV, *n* = 371	
a. Number with follow-up HVL	327/371 (88%)
b. Median time to follow-up HVL, *n* = 327	9.0 months (6.0–12.2)
c. HVL < 100 copies/mL	267/327 (82%)
d. Persistent LLV	41/327 (13%)
e. Unsuppressed HVL	19/327 (6%)
5. Cascade of care for PLHIV with unsuppressed HVL, *n* = 494	
a. No follow-up HVL	69/494 (14%)
b. Virologic failure (VF)	158/425 (37%)
c. Suppressed HVL	267/425 (63%)
i. HVL < 100 copies/mL	216/425 (51%)
ii. LLV	51/425 (12%)
d. Months from date of unsuppressed HVL to date of follow-up HVL, median (IQR), *n* = 425	6.4 months (5.1 – 9.0)
i. ≤ 4 months	102 (24%)
ii. 5—6 months	136 (32%)
iii. 7—9 months	114 (27%)
iv. 10—12 months	40 (9%)
v. ≥ 13 months	33 (8%)
e. Switched to second line ART among suppressed follow up HVL, *n* = 267	
i. Already on second line ART at unsuppressed HVL	109/267 (41%)
ii. Switched to second line ART by follow-up HVL	11/267 (4%)
f. Outcomes for those with VF, *n* = 158	
i. Already on second line ART at follow-up HVL	103/158 (65%)
ii. Of those still on first-line, switched to second line ART	32/55 (58%)
- Months from date of follow-up HVL to date of second line ART switch, *n* = 32	7.7 months (4.7–12.7)
iii. No switch to second line despite VF	23/55 (42%)
-Had a third HVL	16/23 (26%)
i. Unsuppressed HVL	3/16 (19%)
ii. Suppressed HVL	13/16 (81%)
- Months from date of follow-up HVL to date of status (database censor date/LTFU/death), *n* = 23	16.4 months (9.1–25.8)
- ≤ 4 months since follow-up HVL	5/23 (22%)
- Active in care	14/23 (61%)
- LTFU	6/23 (26%)
- Died	2/23 (9%)
- Transfer out	1/23 (4%)

*HVL* HIV viral load, *LTFU* lost to Follow-up

### Cascade after unsuppressed HVL

Among the 494 (12%) PLHIV with unsuppressed HVL, 425 (86%) had a follow up HVL done after a median of 6.4 months (IQR 5.1–9.1). Of those, 267 (63%) were virally suppressed, whereby 216 (51%) had a HVL < 100 copies/ml, 51 (12%) had LLV and 158 (37%) had VF (Fig. 2 and Table 2). Among 69 PLHIV with no follow-up HVL, 10 (15%) were in active care, 41 (59%) had been lost to follow-up (LTFU), 6 (9%) had died and 12 (17%) were transferred to another clinic. Within study duration, the median follow-up time of unsuppressed HVL (among those active-in-care), LTFU, death or transfer to another clinic was 6.1 months (IQR 3.0 -9.8).

Of the 158 PLHIV with VF, 103 (65%) were already on second-line ART at the time of follow-up HVL. Among the 55 PLHIV on first-line ART, 32 (58%) were switched from first- to second-line ART after a median of 7.7 months (IQR 4.7–12.7). Of the 23 PLHIV not switched, 14 (61%) were in active care, 6 (26%) were LTFU, 2 (9%) had died and 1 (4%) had transferred to another clinic. Of the 267 PLHIV who had a first unsuppressed HVL and a suppressed follow-up HVL, 109 (40%) were already on second-line ART at the first unsuppressed HVL measurement and 11 (4%) were switched following the first unsuppressed HVL (Fig. 2 and Table 2).

### Cascade after LLV

Among 371 (9%) PLHIV who had LLV, 327 (88%) had a follow up HVL done after a median of 9.0 months (IQR 6.0–12.1). Of these, 267 (82%) had follow-up a HVL < 100 copies/ml, 41 (13%) had persistent LLV and 19 (6%) were unsuppressed (Fig. 2 and Table 2).

### Cascade after suppressed HVL

Among 3,312 (79%) PLHIV with a HVL < 100 copies/ml, 2,876 (87%) had a follow-up HVL done after a median of 12.0 months (IQR 11.8–13.2). Of those, 2,653 (92%) still had HVL < 100 copies/ml, while 132 (5%) had LLV and 91 (3%) were unsuppressed.

### Turn-around time (TAT)

Among 4,454 PLHIV, 12,512 HVL test results were received, with a median number of HVL tests per person of 3 (IQR 2–3). Only 3,342 (27%) HVL tests were processed within ≤ 14 days as per national guidelines recommendation. The median analytical TAT was 27 days (IQR 14–61), whereby at the on-site laboratory it was 21 days (IQR 13–39) versus 59 days (IQR 27–99) at the referral laboratory (*p* < 0.001; Table 3). The overall TAT– including result provision to the patient – was similar irrespective of the site of testing or unsuppressed HVL results, with a median of 91 days (36–94) and 88 days (28–96) respectively. For those with unsuppressed HVL, only 93 (19%) of follow-up HVL samples were processed in ≤ 14 days and the median analytical TAT was 17 days (IQR 8–31) at the on-site versus 71 days (IQR 35–103) at the referral laboratory (*p* < 0.001). The median TAT until a patient received a result was 4.8 months (IQR 3.2–6.8) and only 102 (24%) had a follow-up HVL within 4 months as per national guidelines.

**Table 3:** Time around time for all PLHIV and those with unsuppressed HVL

**Description**	**Median (IQR)**
1. TAT for all HVL tests	
a. Analytical TAT (date sample taken to date result entered into openMRS database), *n* = 12,512	
i. Processed in ≤ 14 days	3,342 (27%)
ii. Overall, median (IQR)	27 days (14–61)
iii. Sample processed at on-site laboratory, median (IQR)	21 days (13–39)
iv. Sample processed at referral laboratory, median (IQR)	59 days (27–99)
b. Overall TAT (date sample was taken to date participant received results (next clinical visit)), *n* = 8,385	
i. Overall, median (IQR)	91 days (36–94)
ii. Sample processed at on-site laboratory, median (IQR)	91 days (33–93)
iii. Sample processed at referral laboratory, median (IQR)	91 days (76–123)
2. TAT for PLHIV with unsuppressed HVL	
a. Analytical TAT, *n* = 494	
i. Processed in ≤ 14 days	93 (19%)
ii. Overall, median (IQR)	49 days (20–88)
iii. Sample processed at on-site laboratory, median (IQR)	17 days (8–31)
iv. Sample processed at referral laboratory, median (IQR)	71 days (35–103)
b. Overall TAT, *n* = 485	
i. Overall, median (IQR)	88 days (28–96)
ii. Sample processed at on-site laboratory, median (IQR)	85 days (30–95)
iii. Sample processed at referral laboratory, median (IQR)	91 days (74–118)
c. Follow-up HVL TAT (date from unsuppressed HVL result entry into openMRS database to date of follow up HVL, *n* = 425	
i. Overall, median (IQR)	4.8 months (3.2–6.8)
ii. Sample processed at on-site laboratory, median (IQR), *n* = 272	5.2 months (3.4–8.2)
iii. Sample processed at referral laboratory, median (IQR), *n* = 153	3.9 months (3.2–5.8)

*TAT* Turnaround time, *HVL* HIV Viral load

## Discussion

In this study on the HVL testing cascade from a rural site we could show, that during the first three years after implementation of HVL monitoring, testing coverage among PLHIV on ART was high with 95% and viral suppression was 88%. The median analytical TAT of samples processed at the on-site laboratory was one third of the time compared to samples sent to the referral laboratory. The time until clinical management, however, remained long with 90 days irrespective of the testing site. Among PLHIV with an unsuppressed HVL only 24% had a follow up HVL within the 4 months recommended by the National HIV treatment guidelines. Of those with a LLV, 6% became unsuppressed within a year.

Compared to studies from other rural sub-Saharan African sites showing HVL monitoring coverage rates ranging from 64–90% [3,15], our testing coverage of PLHIV in care reached 95% following HVL monitoring roll-out by the National AIDS Control Program and the implementing partner USAID Boresha Afya—after starting with 22% in 2017 [16]. The viral suppression rate of 88% in our study is close to the 91% found in a previous study from our site [17] and to the national level of 87% (84% of males and 89% of females) assessed during the Tanzania HIV Impact Survey (THIS) 2016–17 [18, 19]. While these numbers are close to the third 90% of the 2020 UNAIDS goals [20], a gap remains to reach the 2030 UNAIDS goals of 95% viral suppression of those on ART [21]. Reaching UNAIDS 2030 goals might be facilitated by the roll-out of integrase inhibitors as first line treatment, which only started 2019 in Tanzania. In our study, first available HVL measurements were used for analysis where only 6% were on dolutegravir based regimen and majority in the  Non-Nucleoside Reverse Transcriptase Inhibitor (NNRTI) based regimen. [8, 21].

The overall analytical TAT in this real-life rural sub-Saharan African setting was 27 days and analytical TAT at on-site lab was 21 days which are still notably longer than the 14 days recommended by Tanzanian’s National AIDS Control Programme guidelines [8]. The reason was mostly the high number of samples to be processed, as the on-site lab was also a referral laboratory of neighbouring health centres, and a back-up laboratory of the regional laboratory in case of stockouts of reagents or breakdown of machines. Thus, the on-site lab had at times faced significant backlog of samples with limited staff. Additionally, the manual paper work necessitated by the semi-automated National system contributed to workload. On top, results had again to be manually entered in the electronic patient record system. Furthermore, the on-site lab in 2020 faced a half-year stock out of reagents due to the COVID-19 pandemics. The improvement of the analytical TAT by performing the HVL in an on-site laboratory compared to a referral laboratory has previously been reported by others. Studies from sub-Saharan Africa using similar definitions showed median TATs ranging from 15–67 days [22, 23]. A study from Malawi could show a substantial impact of distance from the laboratory to the clinic on the TAT, suggesting inefficiency of specimen transfer as a factor driving longer turnaround times [24]. This was confirmed by a reduction of the median analytical TAT for HVL results of about 3 times after implementation of testing at the on-site laboratory in our study (from 59 to 21 days. Major reasons are inconsistent transport availabilities, weather conditions affecting road conditions, reagent stockouts, equipment and electricity shutdowns and administrative delays [22–24]. Thus, there is a clear benefit of a molecular laboratory capable of conducting HVL testing located in remote areas with high patient numbers. Additionally, point of care platforms such as GeneXpert can reduce the TAT of the results [25] and thus benefit clinically unstable PLHIV with a same day result for HVL targeted monitoring and clinical decision making. In this study, however the number of GeneXpert tests done was very low and therefore not analysed separately.

Disappointingly, the analytical TAT did not affect the time until the patient received the results (overall TAT). The most important challenge was to track patients and convince them to come to an earlier than planned appointment. Reasons for not coming early indicated by PLHIV were socioeconomic constraints – mostly small-scale farmers and fishermen – with inability to come up for transport costs. A considerable number of PLHIV in our study (835 (19%)) live ≥ 50 kms away from the clinic with poor road infrastructure and are usually prescribed drugs for three months. In addition, the procedures in place to track PLHIV with an unsuppressed HVL as per National HIV treatment guidelines were only partially successful as, even if PLHIV have phones, they frequently are not reachable due to network and electricity shortages. Innovative methods of HVL results feedback to PLHIV such as use of mobile short message services might help in overcoming this challenge [26, 27]. The majority of PLHIV with an unsuppressed HVL had re-suppressed at the follow-up testing (63%), which is an indicator for non-adherence rather than resistance and supports the strategy of enhanced adherence counselling. Etoori et al. showed a re-suppression rate in Swaziland of 62% [9] while Glass et al. reported a lower re-suppression rate of 45% in Lesotho [28]. In our study, interestingly, 65% of PLHIV were already on second line at the time first HVL measurement, indicating previous persistent treatment adherence problems. We reported a similar finding from a study of PLHIV switched to second line treatment in whom 13.1% had VF and no resistance could be documented at the time of switch nor after 6–12 months on second line treatment [29].

Until recently, the WHO guidelines defined an unsuppressed HVL using a threshold of ≥ 1000copies/mL [2]. Discussion on the implications of HVL results between 100 and 999copies/mL was ongoing after increasing evidence of poor outcome in patients with LLV [30–32]. Our study reports 9% frequency of LLV. Data from other sites are quite diverse with some reporting high rates of LLV of 23%—38% frequency [33, 34], while others show similar figures around 2–9% [29, 35, 36].

Only in mid of this year, the WHO has updated its guidelines and newly defines virologic suppression as a HVL < 50copies/ml. VF remains defined as two HVL ≥ 1000copies/ml. The recommendation is to do adherence counselling and retest HVL in all patients with HVL values > 50copies/ml [2].

In our study, 6% of those with LLV progressed to unsuppressed HVL in a median time of 9 months hence these PLHIV may benefit from closer monitoring. High-level resistance to at least two drugs has also been documented among PLHIV with LLV of 84–94%. PLHIV having high-level resistance to at least two drugs of NNRTI-based first-line ART regimen in studies from Lesotho [30, 31].

The strength of our study is the prospective real-life setting large cohort of PLHIV. The limitations of our study were: first, no resistance testing was done to identify the reasons for high HVL in particular, among those with unsuppressed HVL who switched to second line ART before having follow up HVL and thus we could not determine if the decision to switch to second-line ART was justified or not. Second, we could not assess the impact of adherence counselling among those with unsuppressed HVL. Similarly, we could not verify if all who had treatment switch to second line had treatment adherence counselling as per the national’s HIV treatment guidelines. Third, analytical TAT at both on-site and referral laboratories may have been affected by stock out of reagents, breakdown of machines, which may result to huge backlog of samples affecting TAT results.

## Conclusion

Robust HVL monitoring is achievable in rural remote resource limited settings and with reduced turnaround time of viral load results. The majority of PLHIV with VF were already on second-line ART indicating persistent adherence problems, meaning that it is crucial enhanced treatment adherence counselling is done timely and early enough to prevent HIV drug resistance development.

## Data Availability

All data generated and analysed during this study are included in this published article.

## Abbreviations

HVL: HIV viral load
PLHIV: People living with HIV
ART: Antiretroviral therapy
KIULARCO: Kilombero and Ulanga Antiretroviral Cohort
SFRH: St. Francis Referral Hospital
MRRH: Morogoro Regional Referral Hospital
IHI: Ifakara Health Institute
CDCI: Chronic Diseases Clinic of Ifakara
LLV: Low-level viremia
WHO: World Health Organization
VF: Virologic failure
TAT: Turnaround time
IQR: Interquartile range
BMI: Body mass index
OpenMRS: Open medical record system
THIS: Tanzania HIV Impact Survey
NNRTI: Non-Nucleoside Reverse Transcriptase Inhibitor

## References

[1] UNAIDS. United Republic of Tanzania | UNAIDS. Available from: https://www.unaids.org/en/regionscountries/countries/unitedrepublicoftanzania. [Cited 2021 May 9].

[2] World Health Organization (2021). Consolidated guidelines on HIV prevention, testing, treatment, service delivery and monitoring : recommendations for a public health approach.

[3] Lecher S, Williams J, Fonjungo PN, Kim AA, Ellenberger D, Zhang G, et al. Progress with scale-up of HIV viral load monitoring — seven Sub-Saharan African countries, January 2015–June 2016. Morb Mortal Wkly Rep. 2016;65(47):1332-5. 10.15585/mmwr.mm6547a2 27906910

[4] MoHCDEC (2015). National HIV viral load testing guideline to support HIV and AIDS prevention, care and treatment.

[5] WHO. Consolidated guidelines on the use of antiretroviral drugs for treating and preventing HIV infection: recommendations for a public health approach. Geneva: World Health Organization. 2013. 24716260

[6] Pham MD, Romero L, Parnell B, Anderson DA, Crowe SM, Luchters S (2017). Feasibility of antiretroviral treatment monitoring in the era of decentralized HIV care: A systematic review. AIDS Res Ther.

[7] Girdwood SJ, Nichols BE, Moyo C, Crompton T, Chimhamhiwa D, Rosen S (2019). Optimizing viral load testing access for the last mile: Geospatial cost model for point of care instrument placement. PLoS One.

[8] Ministry For Health, Community Development, Gender, Elderly AC, National Aids Control Programme (2019). National Guidelines for The Management Of HIV And AIDS.

[9] Etoori D, Ciglenecki I, Ndlangamandla M, Edwards CG, Jobanputra K, Pasipamire M, et al. Successes and challenges in optimizing the viral load cascade to improve antiretroviral therapy adherence and rationalize second-line switches in Swaziland. J Int AIDS Soc. 2018;21(10). Available from: https://www.ncbi.nlm.nih.gov/pmc/articles/PMC6198167/. [Cited 2021 Apr 19]. 10.1002/jia2.25194 PMC6198167 30350392

[10] Keiser O, Tweya H, Braitstein P, Dabis F, MacPhail P (2010). Mortality after failure of antiretroviral therapy in sub-Saharan Africa. Trop Med Int Heal.

[11] Olivia Keiser, Benjamin H Chi, Thomas Gsponer, Andrew Boulle, Catherine Orrell, Sam Phiri et al. Outcomes of Antiretroviral Treatment in Programmes with and without Routine Viral Load Monitoring in Southern Africa. AIDS. 2011; Available from: https://pubmed.ncbi.nlm.nih.gov/21681057/. 10.1097/QAD.0b013e328349822f PMC3605707 21681057

[12] Letang E, Kalinjuma AV, Glass TR, Gamell A, Mapesi H, Sikalengo G (2017). Cohort profile: The Kilombero and Ulanga Antiretroviral Cohort (KIULARCO) - A prospective HIV cohort in rural Tanzania. Swiss Med Wkly.

[13] Vanobberghen F, Letang E, Gamell A, Mnzava DK, Faini D, Luwanda LB, et al. A decade of HIV care in rural Tanzania: Trends in clinical outcomes and impact of clinic optimisation in an open, prospective cohort. PLoS One. 2017;12(7):e0180983. Available from: 10.1371/journal.pone.0180983. [Cited 2021 May 9]. 10.1371/journal.pone.0180983 PMC5515476 28719610

[14] StataCorp (2017). Stata Statistical Software: Release 15.

[15] Nakalega R, Mukiza N, Kiwanuka G, Makanga-Kakumba R, Menge R, Kataike H (2020). Non-uptake of viral load testing among people receiving HIV treatment in Gomba district, rural Uganda. BMC Infect Dis.

[16] Antelman G, van de Ven R, Mukaminega M, Haule D, van ‘t Pad Bosch J, Somi G (2018). Title: Who does HIV viral load testing reach first? Lessons from Tanzania’s first year of scaling up HIV viral load accessibility.

[17] Ntamatungiro AJ, Muri L, Glass TR, Erb S, Battegay M, Furrer H (2017). Strengthening HIV therapy and care in rural Tanzania affects rates of viral suppression. J Antimicrob Chemother.

[18] Statistics NB of. National Bureau of Statistics - The Tanzania HIV Impact Survey 2016–2017 (THIS) - Final Report. Available from: https://www.nbs.go.tz/index.php/en/census-surveys/health-statistics/hiv-and-malaria-survey/382-the-tanzania-hiv-impact-survey-2016-2017-this-final-report. [Cited 2021 Apr 19].

[19] UNAIDS. United Republic of Tanzania | UNAIDS. Available from: https://www.unaids.org/en/keywords/united-republic-tanzania. [Cited 2021 Apr 19].

[20] UNAIDS. 90–90–90: treatment for all | UNAIDS. Available from: https://www.unaids.org/en/resources/909090. [Cited 2021 Aug 4].

[21] UNAIDS. UNAIDS Issues New Fast-Track Strategy to End AIDS by 2030 - EGPAF. Available from: https://www.pedaids.org/2014/11/20/unaids-issues-new-fast-track-strategy-to-end-aids-by-2030/. [Cited 2021 Apr 16].

[22] Mbiva F, Tweya H, Satyanarayana S, Takarinda K, Timire C, Dzangare J (2021). Long turnaround times in viral load monitoring of people living with HIV in resource-limited settings. J Glob Infect Dis..

[23] Shiferaw MB, Yismaw G (2019). Magnitude of delayed turnaround time of laboratory results in Amhara Public Health Institute, Bahir Dar. Ethiopia. BMC Health Serv Res..

[24] Minchella PA, Chipungu G, Kim AA, Sarr A, Ali H, Mwenda R, et al. Specimen origin, type and testing laboratory are linked to longer turnaround times for HIV viral load testing in Malawi. PLoS One. 2017;12(2). Available from: https://pubmed.ncbi.nlm.nih.gov/28235013/. [Cited 2021 Apr 21]. 10.1371/journal.pone.0173009 PMC5325555 28235013

[25] Nicholas S, Poulet E, Wolters L, Wapling J, Rakesh A, Amoros I (2019). Point-of-care viral load monitoring: outcomes from a decentralized HIV programme in Malawi. J Int AIDS Soc.

[26] Lester RT, Ritvo P, Mills EJ, Kariri A, Karanja S, Chung MH (2010). Effects of a mobile phone short message service on antiretroviral treatment adherence in Kenya (WelTel Kenya1): a randomised trial. Lancet.

[27] Da Costa TM, Barbosa BJP, E Costa DAG, Sigulem D, De Fátima Marin H, Filho AC (2012). Results of a randomized controlled trial to assess the effects of a mobile SMS-based intervention on treatment adherence in HIV/AIDS-infected Brazilian women and impressions and satisfaction with respect to incoming messages. Int J Med Inform.

[28] Glass TR, Motaboli L, Nsakala B, Lerotholi M, Vanobberghen F, Amstutz A (2019). The viral load monitoring cascade in a resource-limited setting: a prospective multicentre cohort study after introduction of routine viral load monitoring in rural Lesotho. PLoS One.

[29] Bircher RE, Ntamatungiro AJ, Glass TR, Mnzava D, Nyuri A, Mapesi H, et al. High failure rates of protease inhibitor-based antiretroviral treatment in rural Tanzania – A prospective cohort study. PLoS One. 2020;15(1). Available from: https://pubmed.ncbi.nlm.nih.gov/31929566/. [Cited 2021 Apr 20]. 10.1371/journal.pone.0227600 PMC6957142 31929566

[30] Labhardt ND, Bader J, Lejone TI, Ringera I, Hobbins MA, Fritz C, et al. Should viral load thresholds be lowered? Revisiting the WHO definition for virologic failure in patients on antiretroviral therapy in resource-limited settings. Med (United States). 2016;95(28). Available from: https://journals.lww.com/md-journal/Fulltext/2016/07120/Should_viral_load_thresholds_be_lowered__.11.aspx. [Cited 2021 Jun 16]. 10.1097/MD.0000000000003985 PMC4956783 27428189

[31] Brown JA, Amstutz A, Nsakala BL, Seeburg U, Vanobberghen F, Vanobberghen (2021). Extensive drug resistance during low-level HIV viraemia while taking NNRTI-based ART supports lowering the viral load threshold for regimen switch in resource-limited settings: a pre-planned analysis from the SESOTHO trial. J Antimicrob Chemother..

[32] Amstutz A, Nsakala BL, Vanobberghen F, Muhairwe J, Glass TR, Achieng B, et al. SESOTHO trial (“Switch Either near Suppression Or THOusand”) - switch to second-line versus WHO-guided standard of care for unsuppressed patients on first-line ART with viremia below 1000 copies/mL: Protocol of a multicenter, parallel-group, open-label, randomized clinical trial in Lesotho, Southern Africa. BMC Infect Dis. 2018;18:76:1–9. 10.1186/s12879-018-2979-y. 10.1186/s12879-018-2979-y PMC5810070 29433430

[33] Brown JA, Amstutz A, Nsakala BL, Seeburg U, Vanobberghen F, Vanobberghen (2021). Extensive drug resistance during low-level HIV viraemia while taking NNRTI-based ART supports lowering the viral load threshold for regimen switch in resource-limited settings: a pre-planned analysis from the SESOTHO trial. J Antimicrob Chemother..

[34] Zhang T, Ding H, An M, Wang X, Tian W, Zhao B (2020). Factors associated with high-risk low-level viremia leading to virologic failure: 16-year retrospective study of a Chinese antiretroviral therapy cohort. BMC Infect Dis..

[35] Delaugerre C, Gallien S, Flandre P, Mathez D, Amarsy R, Ferret S (2012). Impact of low-level-viremia on HIV-1 drug-resistance evolution among antiretroviral treated-patients. PLoS One.

[36] Fleming J, Mathews WC, Rutstein RM, Aberg J, Somboonwit C, Cheever LW (2019). Low-level viremia and virologic failure in persons with HIV infection treated with antiretroviral therapy. AIDS..

